# Clostridium perfringens Sepsis With Fulminant Intravascular Hemolysis: Diagnostic Clues From the Peripheral Blood Film

**DOI:** 10.7759/cureus.111285

**Published:** 2026-06-22

**Authors:** Stamatis J Karakatsanis, Stefanos Vlandos, Charalampos-Vasileios Ntelis, Katerina Frantzeskaki, Anastasia Charalambous

**Affiliations:** 1 Hematology Unit, 3rd University Clinic and Affiliated Laboratory, National and Kapodistrian University of Athens, Medical School, Sotiria General Hospital, Athens, GRC

**Keywords:** clostridium perfringens, hemolysis, intraneutrophilic bacteria, peripheral blood film, septic shock

## Abstract

*Clostridium perfringens (C. perfringens)* bacteremia complicated by massive intravascular hemolysis (MIH) is rare, rapidly progressive, and often fatal even before microbiological confirmation is available. We present the case of an 85-year-old man initially presenting with symptoms suggestive of an acute cardiovascular event. Within hours, however, his condition deteriorated significantly, necessitating intubation and admission to the ICU, while severe hemolysis repeatedly rendered his blood samples unsuitable for analysis. At that time, review of the patient’s peripheral blood (PB) film revealed bacillary forms within a neutrophil, providing a significant, early clue to overwhelming toxin-mediated bacterial sepsis, later confirmed to be due to *C. perfringens*, which, despite broad-spectrum antibiotic treatment and intensive supportive care, led to the patient’s death within hours of ICU admission. This report highlights the importance of urgent PB film review in septic patients with otherwise unexplained hemolysis, in order to enable a timely and effective antibiotic regimen, optimized supportive care, and assessment for source control when possible.

## Introduction

*Clostridium perfringens (C. perfringens)* is an anaerobic, Gram-positive, spore-forming bacillus that is most commonly associated with food poisoning and gas gangrene, but only rarely causes bloodstream infections [1]. When bacteremia does occur, it carries a reported mortality of 27-58%, which increases sharply to 70-100% when massive intravascular hemolysis (MIH) develops, a complication seen in approximately 7-15% of *C. perfringens* bacteremia cases and is often fatal within hours of onset [2-5]. In a pooled analysis of 50 reported cases since 1990, the median time from presentation to death was only 9.7 hours (range: 0-96 hours), underscoring the fulminant nature of this entity [3]. The hemolytic process is driven primarily by α-toxin (phospholipase C, CPA), which hydrolyzes membrane phospholipids of erythrocytes, activates platelets, injures the microvasculature, and triggers disseminated intravascular coagulation, shock, and multiorgan failure [2,4,6-8]. Common portals of entry include the hepatobiliary tract, gastrointestinal tract (including occult colonic malignancy), and skin/soft-tissue wounds, particularly in elderly or immunocompromised hosts [3,9]. Because blood cultures take an average of approximately 17 hours to become positive and clinical features are non-specific, early bedside diagnostic indicators are essential for timely recognition and management [4].

## Case presentation

An 85-year-old man presented to the Emergency Department (ED) with acute-onset dyspnea and retrosternal chest discomfort of approximately a one-hour duration. His medical history included Parkinson’s disease, arterial hypertension, and dyslipidemia. He had no known drug allergies, had quit smoking more than 20 years ago, and reported only occasional alcohol use. His regular medications included levodopa/benserazide, rasagiline, ramipril, simvastatin, omeprazole, and aspirin.

Upon his arrival at the ED, the patient was alert, oriented, and able to communicate, yet tachypneic and hypoxic on room air (SpO₂ 92% by pulse oximetry). The rest of the patient's vital signs and capillary refill time were normal. Cardiac auscultation revealed normal first and second heart sounds with a grade 2/6 systolic murmur along the right parasternal border. Respiratory examination showed fine late-inspiratory crackles at both lung bases, more prominent on the right. The patient's abdomen was soft and non-distended, with mild right upper-quadrant tenderness and normal bowel sounds. Peripheral pulses were palpable and symmetrical, with no edema. An abrasion was noted on the patient's right lower leg, with central crust and surrounding violaceous-to-brownish discoloration, as well as perilesional ecchymosis, without signs of acute local inflammation, reportedly from a minor injury that had occurred one to two weeks before presentation in the patient's own garden.

**Figure 1:**
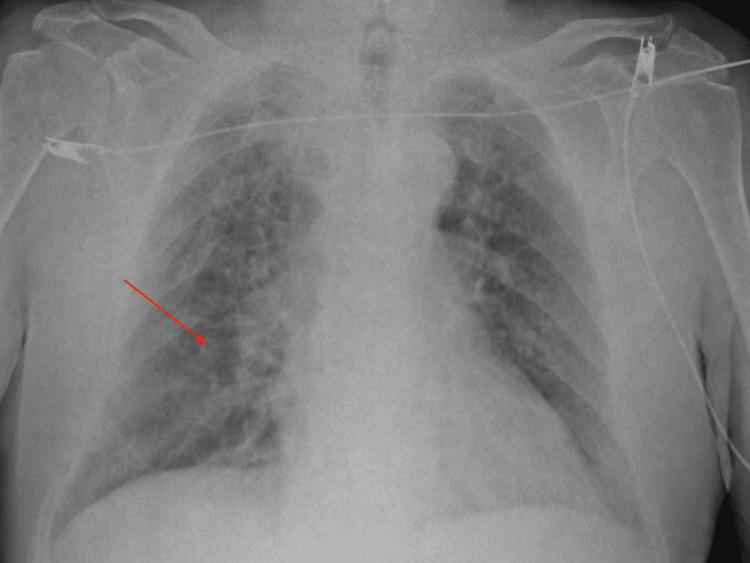
Chest radiograph consistent with cardiac failure Portable anteroposterior chest radiograph showing features in keeping with cardiac failure, including pulmonary vascular congestion/interstitial oedema. The red arrow indicates the perihilar/lower-zone pulmonary congestion, representing the principal radiographic area of interest

The initial working diagnosis was acute coronary syndrome or acute cardiac decompensation. His electrocardiogram and high-sensitivity troponin results were not diagnostic of acute coronary syndrome, yet the patient's chest radiograph was suggestive of cardiac failure (Figure 1), and the first bedside echocardiographic assessment suggested a left ventricular ejection fraction of about 50% with possible lateral-wall hypokinesia.

**Figure 2:**
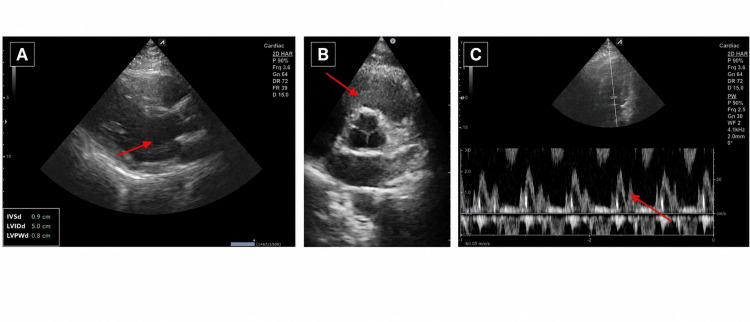
Transthoracic echocardiography (A) Parasternal long-axis view showing a normal-sized left ventricle with preserved systolic function; the arrow indicates the left ventricular cavity/myocardium used for assessment. (B) Echocardiographic view demonstrating a non-dilated right ventricle with preserved longitudinal systolic function; the arrow indicates the right ventricular area of interest. (C) Pulse-wave Doppler assessment of the left ventricular inflow tract. The arrow indicates normal E and A values, with a normal E/A ratio and no diastolic dysfunction of the left ventricle. Continuous-wave Doppler across the tricuspid valve showed mild tricuspid regurgitation, with an estimated right ventricular systolic pressure of approximately 35 mmHg. Overall, the examination showed a left ventricular ejection fraction of approximately 55%, no major valvular disease, and no pericardial effusion

The patient was therefore admitted to the Coronary Care Unit for monitoring, repeat echocardiography, and consideration of coronary angiography. Formal echocardiography later (Figure 2) showed a normal-sized left ventricle with preserved systolic function, no major valvular disease, mild tricuspid regurgitation with an estimated right ventricular systolic pressure of approximately 35 mmHg, a non-dilated right ventricle with a tricuspid annular plane systolic excursion (TAPSE) value of 23 mm, and no pericardial effusion.

Shortly after the patient’s admission, the Transfusion Medicine Laboratory reported that his blood samples were grossly hemolyzed and could not be reliably processed. The patient then developed a fever of up to 38.9 °C and worsening right upper-quadrant pain, while passing two loose stools. As soon as the patient’s initial laboratory results became available, they were notable for anemia, marked leukocytosis, hemolysis, prolonged prothrombin time (PT) and activated partial thromboplastin time (aPTT), elevated inflammatory markers, acute kidney injury and hepatocellular injury, and markedly elevated ferritin levels, while D-dimer levels could not be measured due to pre-analytical errors from the patient’s severely hemolyzed samples. Right upper-quadrant ultrasonography showed only mild gallbladder distension and biliary sludge, and empirical piperacillin-tazobactam and vancomycin were initiated after surgical assessment.

**Figure 3:**
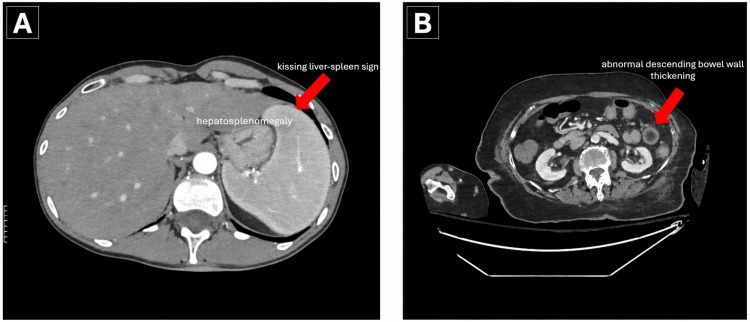
Contrast-enhanced axial abdominal CT showing hepatosplenomegaly (A) and colitis (B) (A) Marked hepatosplenomegaly with close apposition of the enlarged liver and spleen, producing the “kissing liver–spleen” sign; the red arrow indicates the area of liver–spleen contact. (B) Segmental abnormal wall thickening of the descending colon, compatible with colitis; the red arrow indicates the affected colonic segment CT: computed tomography

Despite these measures, the patient's condition deteriorated rapidly. Over the next few hours, he developed increasing tachypnea, hypoxemia, tachycardia, and hemodynamic instability. Oxygen therapy was escalated, followed by intubation, vasopressor support, and ICU admission due to presumed septic shock, while a CT of the abdomen showed hepatosplenomegaly and colitis (Figure 3).

**Figure 4:**
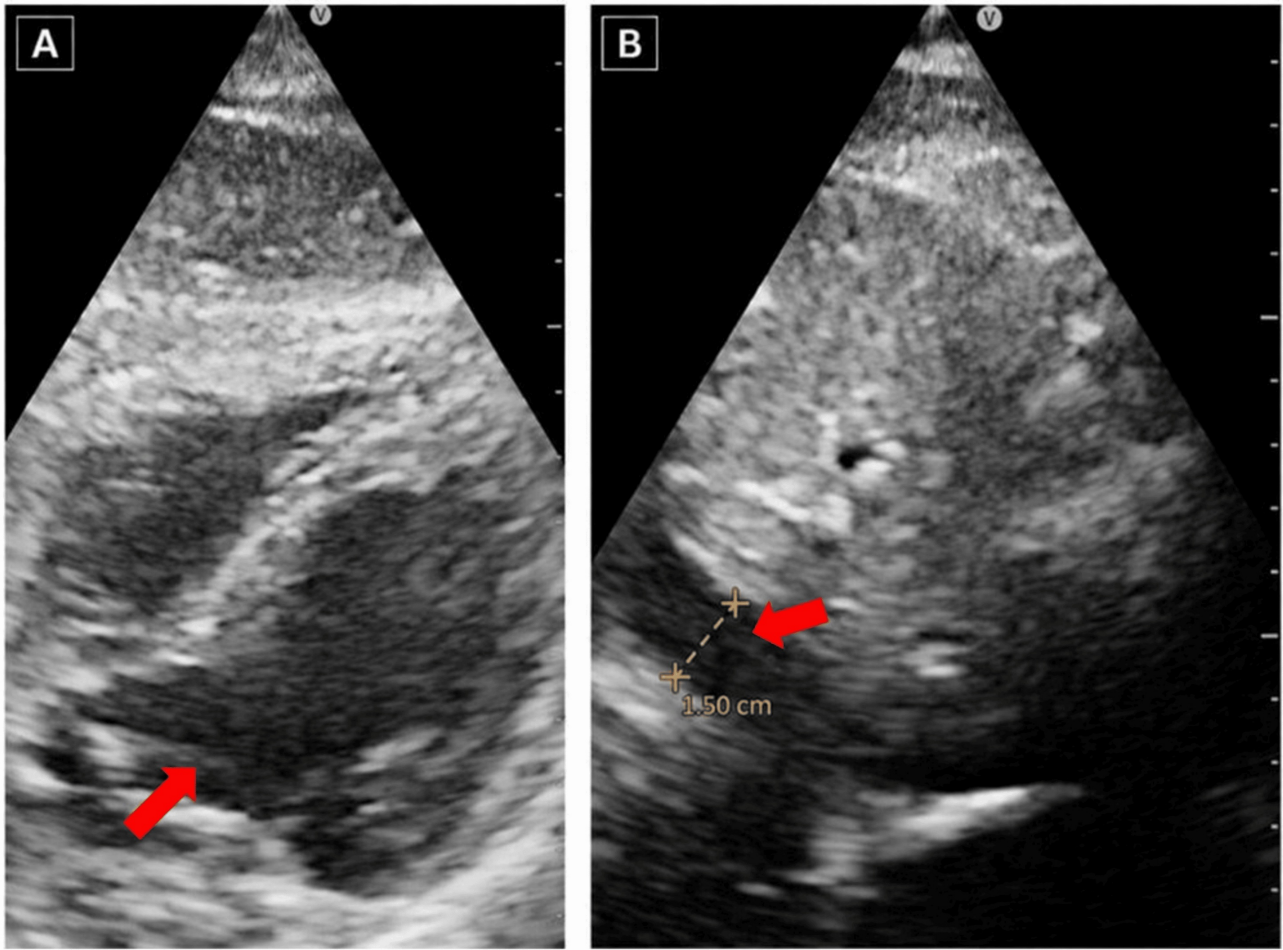
Transthoracic echocardiography demonstrating left ventricular systolic dysfunction and normal inferior vena cava dynamics (A) Echocardiographic view showing global left ventricular hypokinesia; the red arrow indicates the left ventricular cavity and myocardium assessed for global systolic dysfunction. (B) Subcostal inferior vena cava view showing a normal inferior vena cava diameter, measured at approximately 1.50 cm, with preserved respiratory variation; the red arrow indicates the measured inferior vena cava segment

A third cardiac ultrasound at that stage showed global left ventricular hypokinesia and a normal inferior vena cava with preserved respiratory variation, findings consistent with an evolving systemic illness rather than a primary cardiac disease (Figure 4).

**Figure 5:**
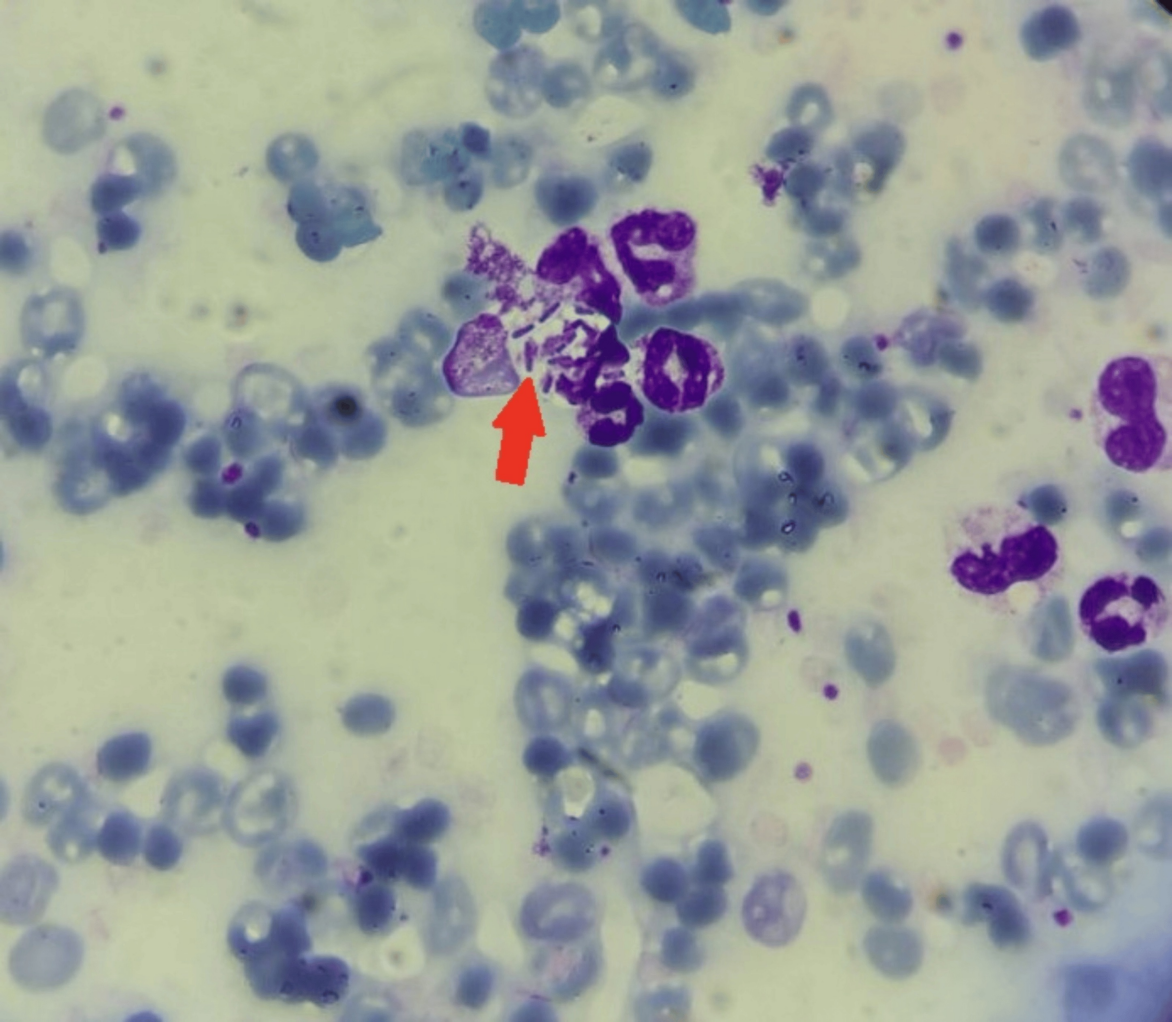
Review of PB film in fulminant Clostridium perfringens sepsis The smear shows marked hemolytic changes, along with bacillary forms phagocytosed within a neutrophil (arrow). In the appropriate clinical context, the combination of massive intravascular hemolysis and intraneutrophilic bacilli is a critical early clue to clostridial sepsis before culture confirmation PB: peripheral blood

Six hours later, the patient's hemoglobin value had dropped from 10.9 to 6.0 g/dL (reference range: 13-17 g/dL) due to brisk hemolysis, worsening coagulopathy, and progressive renal and hepatic injury. Thus, empirical methylprednisolone 1 g was added, and a differential diagnosis was discussed while supportive care was optimized. At that point, review of the patient's peripheral blood (PB) film by the Hematology Department revealed bacillary forms within a neutrophil (Figure 5), strongly supporting a diagnosis of overwhelming bacterial sepsis with massive intravascular hemolysis rather than another type of hemolytic anemia.

In 12 hours, the patient’s hemoglobin had declined further to 3.6 g/dL, the white blood cell (WBC) count had risen to 34.7 × 10^3^/μL, INR had increased to 6.12, LDH was 9,550 U/L, total bilirubin was 8.4 mg/dL, and procalcitonin was 16.79 ng/mL (Table 1). A broad microbiological work-up was performed (including molecular testing, cultures, and urinary antigen tests), and antibiotic treatment was then modified to piperacillin/tazobactam plus moxifloxacin. Despite the intensive ventilatory and hemodynamic supportive measures, the patient died within hours of his ICU admission from refractory septic shock, fulminant hemolysis, and multiorgan failure. Blood cultures later returned positive for* C. perfringens*.

**Table 1:** Serial laboratory trajectory of the patient during the first 12 hours ALT: alanine aminotransferase; aPTT: activated partial thromboplastin time; AST: aspartate aminotransferase; CK-MB: creatine kinase myocardial band; CPK: creatine phosphokinase; CRP: C-reactive protein; dL: deciliter; L: liter; FEU: fibrinogen-equivalent units; g: grams; h: hours; hs-cTnT: high-sensitivity cardiac troponin T; INR: international normalized ratio; LDH: lactate dehydrogenase; mg: milligram; mmol: millimole; ng: nanogram; PT: prothrombin time; s: seconds; SI: International System of Units; t: time from presentation/admission; U: units; WBC: white blood cell; μg: microgram; μL: microliter; μmol: micromole Laboratory units are shown as reported in the source material where available. D-dimer testing could not be completed because of gross sample hemolysis

Variable	Reference range	t = 0	t = 6 h	t = 12 h
Hemoglobin, g/dL	Male: 14–17	10.9	6	3.6
WBC count, ×10^3^/µL	5.0–10.0	28.25	29.29	34.7
WBC differential count, neutrophils/lymphocytes/monocytes/other, %	40–75/20–45/1–9/approximately 1–5	89/5/3/0.5	89/6/3/1	87/8/2/1
Platelets, ×10^3^/µL	150–400	217	237	128
INR	0.8–1.1	2.34	3.27	6.12
PT, s	11–12.5	29	40	72
aPTT, s	30–40	71	78	144
Fibrinogen, mg/dL (g/L)	200–400 (2.0–4.0)	201 (2.01)	216 (2.16)	229 (2.29)
D-dimer	Usually <0.5 mg/L FEU or <500 µg/L FEU; assay-dependent	Not measurable because of hemolysis	Not measurable because of hemolysis	Not measurable because of hemolysis
CRP, mg/dL (mg/L)	<1.0 (<10)	6.01 (60.1)	6.65 (66.5)	10.09 (100.01)
Procalcitonin, ng/mL (µg/L)	0.00–0.24	5.12	6.23	16.79
Urea, mg/dL (mmol/L)	15–45 (2.5–7.5)	84 (14)	92 (13.7)	104 (32.3)
Creatinine, mg/dL (µmol/L)	Male: 0.6–1.2 (53–106)	1.8 (159)	2.1 (186)	2.7 (239)
LDH, U/L	100–190	8,218	8,258	9,550
Total/direct bilirubin, mg/dL (µmol/L)	Total: 0.3–1.0 (5.1–17); direct: 0.1–0.3 (1.7–5.1)	3.5/0.7 (60/8)	6.5/1.2 (111/21)	8.4/1.3 (144/22)
AST/ALT, U/L	AST: 0–35; ALT: 4–36	567/122	834/183	1,468/669
CPK/CK-MB, U/L	CPK: 20–200; CK-MB: usually <25 U/L or <5% of total CPK, assay-dependent	842/56	1,087/not available	628/not available
Ferritin, ng/mL (µg/L)	Male: 30–300	13,640	15,800	31,627
Troponin, ng/L	Male: ≤22	23	12	87

## Discussion

Primarily, this case report highlights the rapidly progressive course of illness in an older patient and the diagnostic difficulty associated with *C. perfringens* sepsis. Second, the detection of intraneutrophilic bacilli on the PB film - together with markedly hemolyzed plasma, a rapid and profound decline in hemoglobin, and microspherocytosis - was critical for identifying overwhelming bacterial sepsis with toxin-mediated intravascular hemolysis well before culture confirmation [4,5,10].

Hence, this case report underscores the importance of PB film review in patients with septic shock and rapidly progressive hemolysis, particularly since the patient's initial presentation was potentially misleading: his complaints of dyspnea and chest pain, along with the abnormal chest radiograph and initial bedside echocardiographic findings, reasonably prompted concern for a cardiovascular etiology requiring cardiac monitoring. Within hours, however, the syndrome evolved into fulminant sepsis with severe hemolysis, coagulopathy, acute kidney and liver injury, and shock. This nonspecific early clinical picture aligns with existing literature, which consistently describes *C. perfringens* bacteremia as highly variable and often mistaken for cardiac, gastrointestinal, or hepatobiliary conditions, thereby delaying anti-anaerobic therapy and source-control decisions [1,9]. In this context, the PB film finding offered greater diagnostic insight into the underlying pathology than initial laboratory results, symptoms, or clinical examination findings.

*C. perfringens* is an anaerobic, Gram-positive, spore-forming bacillus most commonly encountered as a cause of food poisoning and gas gangrene, with bacteremia representing an uncommon but disproportionately lethal manifestation [1]. Reported in-hospital mortality of C. perfringens bacteremia ranges from 27% to 58%, but increases to 70% to 100% when MIH occurs, a complication that affects approximately 7% to 15% of bacteremic episodes [2,8,10]. In a pooled analysis of 50 cases published since 1990, the median time from clinical presentation to death was only 9.7 hours (range: 0 to 96 hours), with most patients dying within the first 24 hours after recognition [8]. The clinical course in our patient, namely death within hours of ICU admission despite escalating ventilatory, hemodynamic, and antimicrobial support, is therefore consistent with the published natural history of fulminant clostridial sepsis with MIH [8,9].

In the present case, colitis and right upper-quadrant symptoms suggested a possible gastrointestinal or hepatobiliary source, whereas the recent right lower-leg abrasion represented another plausible soft-tissue entry point. The available data did not allow this to be assigned with certainty. These three potential portals of entry, namely the hepatobiliary tract (including cholangitis, liver abscess, and biliary stenting), colonic mucosa (particularly in the setting of occult malignancy or ischemic colitis), and skin or soft-tissue breaches, together account for the majority of *C. perfringens* bacteremic episodes, especially in elderly hosts and patients with diabetes, malignancy, or immunosuppression [7,8].

The biological basis of *C. perfringens* hemolysis is toxin-mediated and involves the disruption of the membranes of the erythrocytes, microvascular injury, platelet activation, shock, and disseminated intravascular coagulation [2-5]. Mechanistically, the dominant virulence factor is α-toxin (phospholipase C, CPA), produced by toxinotype A and F strains, which hydrolyses phosphatidylcholine and sphingomyelin in the erythrocyte membrane. This generates the characteristic microspherocytes, "ghost cells" and dehemoglobinized erythrocytes seen on the PB film, while simultaneously activating platelets and endothelium to precipitate disseminated intravascular coagulation and shock [9,10]. In such cases, marked sample hemolysis should alert the laboratory staff to the possibility of clostridial sepsis or another severe bacterial infection, so that they can notify the clinical care team to promptly examine for intracellular bacilli, given the particularly poor prognosis [2-6,10].

Importantly, blood cultures in *C. perfringens* bacteremia flag positive comparatively quickly - on average within approximately 17 hours - yet even this is often too late given a median time to death of less than 10 hours in MIH [8,9]. The PB film, therefore, occupies a unique diagnostic niche: it can be examined within minutes of recognizing gross sample hemolysis and provides actionable information well before culture-based confirmation [6,9,10]. In our patient, intraneutrophilic bacilli on Giemsa-stained smear, together with grossly hemolyzed plasma and a precipitous fall in hemoglobin, formed a near-pathognomonic triad that should redirect empirical therapy toward broad anaerobic coverage without delay.

This report has an important practical clinical implication: in a patient with sepsis, grossly hemolyzed samples and a rapid drop in their hemoglobin value, urgent Gram stain, cultures, and immediate expert review of the PB film are essential. The finding of intraneutrophilic bacilli, even before culture confirmation, should prompt specific consideration of clostridial sepsis, rapid initiation of broad anaerobic antibiotic treatment, as well as intensive supportive care and source control if feasible [8-10]. Although evidence for adjunctive measures such as hyperbaric oxygen therapy, exchange transfusion, or surgical debridement remains limited to case reports and small series, the consistent message from the literature is that outcome is determined far more by the speed of recognition than by any specific therapeutic intervention once MIH is established [8,9].

## Conclusions

Fulminant *C. perfringens *bacteremia with MIH remains one of the most rapidly lethal infections encountered in modern critical care, with mortality approaching 100% and median survival measured in hours once hemolysis is established. The diagnostic window between clinical deterioration and death is therefore narrower than the time required for conventional microbiological confirmation, and clinicians cannot afford to wait for culture results before acting. This report illustrates that, when the constellation of unexplained sepsis, grossly hemolyzed plasma, a precipitous fall in hemoglobin, and disproportionate coagulopathy is encountered, expert PB film review should be requested as an emergency investigation alongside Gram stain and cultures. The visualization of intraneutrophilic bacilli - a finding that can be obtained within minutes - should be treated as a clinical emergency, prompting immediate broad-spectrum anaerobic antibiotic coverage, aggressive resuscitation, and active pursuit of a source amenable to control. Heightened awareness of this near-pathognomonic morphological triad, together with close real-time communication between the laboratory and the bedside team, offers the most realistic opportunity to alter the otherwise uniformly grim outcome of clostridial sepsis with MIH.
